# LbL-assembled gentamicin delivery system for PMMA bone cements to prolong antimicrobial activity

**DOI:** 10.1371/journal.pone.0207753

**Published:** 2018-12-13

**Authors:** Yazan Al Thaher, Lirong Yang, Steve A. Jones, Stefano Perni, Polina Prokopovich

**Affiliations:** 1 School of Pharmacy and Pharmaceutical Science, Cardiff University, Cardiff, United Kingdom; 2 University Hospital Llandough, Cardiff & Vale University Health Board, Vale of Glamorgan, Wales, United Kingdom; Brandeis University, UNITED STATES

## Abstract

**Introduction:**

Antibiotic-loaded poly(methyl methacrylate) bone cements (ALBCs) are widely used in total joint replacement (TJR), for local delivery of antibiotics to provide prophylaxis against prosthetic joint infections (PJI). One of the shortcomings of the current generation of ALBCs is that the antibiotic release profile is characterized by a burst over the first few hours followed by a sharp decrease in rate for the following several days (often below minimum inhibitory concentration (MIC)), and, finally, exhaustion (after, typically, ~ 20 d). This profile means that the ALBCs provide only short-term antimicrobial action against bacterial strains involved PJI.

**Rationale:**

The purpose of the present study was to develop an improved antibiotic delivery system for an ALBC. This system involved using a layer-by-layer technique to load the antibiotic (gentamicin sulphate) (GEN) on silica nanoparticles, which are then blended with the powder of the cement. Then, the powder was mixed with the liquid of the cement (NP-GEN cement). For controls, two GEN-loaded brands were used (Cemex Genta and Palacos R+G). Gentamicin release and a host of other relevant properties were determined for all the cements studied.

**Results:**

Compared to control cement specimens, improved GEN release, longer antimicrobial activity (against clinically-relevant bacterial strains), and comparable setting time, cytocompatibility, compressive strength (both prior to and after aging in PBS at 37 ^o^C for 30 d), 4-point bend strength and modulus, fracture toughness, and PBS uptake.

**Conclusions:**

NP-GEN cement may have a role in preventing or treating PJI.

## Introduction

Total joint replacements (TJRs), such as hip and knee replacements, are increasingly used worldwide because of the “graying” of population and risk factors such as obesity [1, 2]. More than 1 million total hip and knee replacement (THJRs and TKJRs) are performed annually in the United States [3] and >180000 such replacements were performed in the UK during 2016 [4]. Periprosthetic joint infections (sometimes also called prosthetic joint infections (PJI)) are a serious adverse event that not only reduces success rate of TJRs, resulting in the need for revision surgery, but, sometimes, leads to patient death [5, 6]. The treatment of such infections is complex, usually involving aggressive surgical intervention (debridement) and long antimicrobial therapy, which negatively impacts a patient’s quality of life and places a huge burden on a health care system [7, 8].

In many THJRs and TKJRs, the anchoring agent is a PMMA bone cements bed and antibiotic-loaded PMMA bone cements (ALBCs) are used both as a prophylaxis against as well as treatment for PJI [9, 10]. The most common antibiotics employed in commercially available ALBC are aminoglycosides, in particular gentamicin sulphate [11]. Gentamicin sulphate is thermostable and can withstand the exothermic polymerization reaction of PMMA bone cement. It is also available in powder form which makes it ideal for mixing with the cement powder as a premixed or off-label formulation [12].

There are many concerns about ALBCs, in particular, the release profile of the antibiotic [13, 14]. Specifically, the profile comprises a burst for the first few hours after surgery, followed by slow release below inhibitory levels within a few days [15]. This release profile does not provide long-term prophylaxis against early- and delayed- stage infections (defined as infections that are contracted during the first 24 h-1 wk and after 1 mo, respectively) [16–20]. In addition, <10% of the antibiotics is released, with the rest trapped within the hydrophobic PMMA matrix [18, 19]. Thus, there is scope for development of ALBCs that display improvements in the aforementioned features of antibiotic release without compromise of other properties of the curing and cured cement.

Layer-by-layer assembly (LbL) comprises deposition of alternatively oppositely-charged polyelectrolytes on different substrates, allowing control of the composition and thickness of the assembly at nanoscale level in a reproducible manner [21, 22]. This coating process is simple, low-cost, scalable and does not utilize harsh organic conditions, as it involves mild aqueous solutions. Because of these advantages, LbL has numerous applications in drug delivery [23–25]. Nanoparticles have been extensively explored, and successfully applied, as drug carriers for antibiotics and other drugs [26, 27]. Among nano-carriers, silica nanoparticles are preferred because of their unique physicochemical properties, biocompatibility and low cost [28]. Silica nanoparticles have a large surface area-to-mass ratio and are amenable to structural or functional modification because of a silanol-containing surface [23, 29, 30].

We propose using gentamicin LbL-loaded silica nanoparticles to prolong GEN release for several weeks (4-6 weeks) to provide prophylaxis from postsurgical PJIs. We intend using GEN LbL loaded silica nanoparticles (NPs) previously characterised [31], developed using poly-beta-amino-ester (PBAE) as the polycation and alginate as the polyanion, to prolong and increase the release of GEN from a bone cement. NPs were made of 10 quadruple layers of the sequence (alginate, gentamicin, alginate, PBAE) and have a diameter of about 50 nm. Many approaches to extend antibiotic release from nanoparticles have been tested and it has been shown that LbL is very effective [30].

We used two popular cement brands (Cemex and Palacos R) and determined a large collection of properties of each of these brands both as-received and after incorporation of the nanoparticles. These properties were setting time, gentamicin release, efficacy of released gentamicin against various bacteria (in their planktonic form) commonly causing PJI, mechanical properties (compression, bending and fracture toughness), cytocompatibility toward human osteoblast cells (MTT, LDH, cell calcium production, and cytoskeletal morphology), and PBS uptake.

## Materials and methods

### Chemicals

Triton X-100, tetraethyl orthosilicate (TEOS), 3-aminopropyltriethoxysilane (APTS), sodium alginate, gentamicin sulphate, sodium acetate trihydrate, phosphate buffer solution (PBS) tablets, o-phthaldialdehyde reagent solution (OPA), piperazine, and 1,6 hexanediol diacrylate were purchased from Sigma-Aldrich, UK. Cyclohexane, n-hexanol, ammonium hydroxide (35%), diethyl-ether, di-chloro methane (DCM), ethanol, methanol, glacial acetic acid, and iso-propanol were purchased from Fisher Scientific, UK. All reagents were stored according to manufacturer’s guidelines and used as received. The bone cements brands used were Cemex (Tecres S.p.A., Italy) and Palacos R (Heraeus Medical GmbH, Germany).

Acetic acid-sodium acetate buffer (0.1 M, pH 5) was prepared mixing sodium acetate trihydrate (CH_3_COONa·3H_2_O) (0.1 M) and acetic acid (CH_3_COOH) (0.1 M) solutions 3:7 and stirred, with pH checked and adjusted in the range 5.0 ± 0.1. PBS solution (pH 7.4) was prepared according to manufacturer’s guidelines.

### Poly-beta-amino-ester synthesis

Amino-terminated poly(β-amino ester)s (PBAEs) were synthesized as described in Al Thaher et al. [31] by mixing 1,6 hexanediol diacrylate and piperazine in a 1:1.1 ratio in DCM at a concentration of 5 ml of DCM and 3.7 mmol of acrylate. The polymerization was performed under stirring at 50°C for 48 hours. PBAEs were precipitated through pouring the reaction mixture in about 10 times the volume of diethyl-ether under vigorous mixing, after which the solvents were removed under vacuum.

### Nanoparticle preparation

#### Amino functionalised silica nanoparticles synthesis

Silica nanoparticles functionalised with amine groups (SiO_2_-NH_2_) (NPs) were prepared through the Stöber method in a one-pot synthesis by hydrolysis of TEOS in reverse micro-emulsion [32] and subsequent functionalization. In a typical synthesis, Triton X-100 (17.7 g) was mixed with 16 mL of n-hexanol, 75 mL of cyclohexane, and 4.8 mL of deionised water under vigorous stirring. Once the solution was transparent, 600 μL of NH_4_OH (29.6%) were added. The solution was subsequently sealed, after stirring for 20 minutes 1 ml of TEOS was added and stirring continued for further 24 h. The surface of the silica nanoparticles was functionalized with amino groups by adding 50 μl of APTS to the micro-emulsion under stirring and incubating for further 24 hours. The NPs were recovered by adding ethanol (200 ml) to break the microemulsion and centrifuging at 14000 rpm for 10 min (LE-80K, Ultracentrifuge, Beckman Coulter, UK) at 20°C (35280 g). The NPs were vigorously washed three times with deionized water. Finally, the washed nanoparticles were left to dry at room temperature in a fume hood for 24 h.

#### Layer by Layer (LbL) coating technique

The NPs were layered with a repeating sequence of the polyelectrolytes (sodium alginate, PBAE) and the antibiotic (GEN), with the final assembly hereafter referred to as NP-GEN, as described in Al Thaher et al. [31].

The sequence was composed of multiples units of four layers (quadruple layer). Each quadruple layer was made of sodium alginate, GEN, sodium alginate and PBAE. The following concentrations of polyelectrolytes and drug in acetic acid-sodium acetate buffer pH 5 were used in LbL: sodium alginate (2 mg/mL), GEN (10 mg/mL), and PBAE (2 mg/mL).

The dried amino functionalized silica nanoparticles (~1 g) were placed in a test tube and dispersed in 20 mL of sodium alginate solution and stirred for 10 minutes. Then, the dispersed nanoparticles were centrifuged to pellet nanoparticles. After that, the supernatant was removed and replaced with 20 mL of fresh acetic acid-sodium acetate buffer as a washing step to remove traces of the un-layered polyelectrolyte. Then, the buffer was centrifuged and removed, leaving the washed nanoparticles ready for next layer. 10 mL of GEN were stirred with the nanoparticles for 10 min, centrifuged, and washed again with buffer. Next, sodium alginate solution was layered again and washed. Finally, 20 mL of the PBAE solution were used to layer the fourth layer and washed, completing the first quadruple layer. This sequence was repeated to build up 10 quadruple layers of NP-GEN.

### Bone cement preparation

Four bone cement formulations were used: as-received Cemex Genta (Cemex Genta), Cemex with incorporated NP-GEN (Cemex-NP-GEN), as-received Palacos R+G (Palacos R+G) and Palacos R with incorporated NP-GEN (Palacos- NP-GEN). For the compositions of these cements, see **Table 1**. Calculations for the nanoparticle containing bone cements were based on the loading efficiency (30% w/w) previously determined [31], to have equal amounts of gentamicin between commercial and nanoparticles loaded bone cements.

**Table 1:** Composition of the test bone cements (in g*).

	Palacos R+G	Palacos-NP-GEN	Cemex Genta	Cemex-NP-GEN
**Liquid component**	18.80	18.80	13.30	13.30
Methyl Methacrylate	18.4	18.4	13.17	13.17
N-N Dimethyl-p-Toluidine	0.4	0.4	0.12	0.12
Hydroquinone / ppm	60.00	60.00	75.00	75.00
**Powder Component**	40.84	42.80	40.00	43.95
Polymethyl Methacrylate			33.12	33.12
Poly(Methyl Methacrylate, Methyl acrylate)	33.6	33.6	0.0	0.0
Chlorophyll	0.008	0.008	0.0	0.0
Benzoyl peroxide	0.3	0.3	1.20	1.20
BaSO_4_	0.0	0.0	4.00	4.00
Zirconium dioxide	6.1	6.1	0.0	0.0
Gentamicin sulphate	0.84	0.0	1.69	0.0
NP-GEN	0.0	2.80	0.0	5.63

* Except for hydroquinone

Cement constituents were stored at recommended conditions (20-25ºC for the powder and 8-15 ºC for the liquid in the dark) and conditioned to room temperature (23ºC) for 2 hours before mixing [33]. Powder components were sifted before weighing and mixed thoroughly. The resulting powder mixture and the cement liquid were hand mixed in a polypropylene bowl with a polypropylene spatula for 1 min and then the dough was poured into polytetrafluoroethylene (PTFE) moulds with sizes and configurations appropriate for specimen preparation for the various cement properties to be determined. After pouring the cement dough into a mould, two steel endplates covered in PTFE film were clamped at both ends of the moulds. After 2 hours, the specimens were pushed out of the mould using a steel rod and allowed to cure for 24±2 h at 23±1 ºC. Then, the specimens were sanded down to the correct dimensions using 320 grit SiC papers.

### Rheology testing

These tests were performed using a rheometer (Anton Paar MRC702, Anton Paar Ltd., UK), equipped with 6 mm diameter circular flat plates (separation distance = 1 mm). During a test, a sinusoidal stress (σ), of small amplitude (σ_0_) and frequency (ω), was applied to a cement dough, which had been placed on the lower plate. The tests were started very shortly thereafter, with the applied strain (ε(t)) and frequency being 0.1% and 1 Hz, respectively.

Note that (i) in this test, the complex modulus of the cement (G*) is the ratio of the response stress (σ(t)) to ε(t); (ii) G* = G´ + iG”, where G´ and G” are the storage modulus and loss modulus of the cement, respectively; (iii) the ratio (G”/G´) = tan δ, where δ is the phase angle between the stress and the strain signals; (iv) the test ran continuously from when the dough was placed on the plate until it polymerized; thus, tan δ was obtained continuously; and (v) the setting time of the cement is the is time the maximum of the tan δ-versus-time curve occurs.

Each sweep experiment was carried out on three independently prepared cement samples [34], and results are presented as mean and standard deviation.

### Gentamicin release quantification

Cylindrical specimens (diameter and height = 6 mm and 10 mm respectively) were prepared using an appropriate PTFE mould. The specimens were incubated in 3 mL PBS buffer (pH 7.4) at 37ºC. The buffer was replaced each day in order to attain sink condition, where the concentration of released gentamicin is negligible in comparison to its saturation solubility. The specimens were stored in a refrigerator (2-8 ºC) for no more than 3 d prior to analysis. For each of the cements, 6 specimens were tested; when the bone cements were mixed with nanoparticles these were independent batches.

The amount of GEN released from the nanoparticles in the buffer was quantified through fluorescence spectroscopy using o-phthaldialdehyde reagent [35] that reacts with the amino groups of GEN producing a fluorogenic product. 70 μL of buffer containing antibiotic were mixed with 70 μL of iso-propanol and 70 μL of OPA reagent solution in a black 96-wells plate. After 30 min at room temperature in the dark, the fluorescence was determined (Ex = 340 nm and Em = 450 nm) with a fluoroscan (FLUOROstar Optina, BMG Labtech). At each time-point, gentamicin concentration was calculated. Six independent solutions with gentamicin concentrations ranging from 0 μg/mL to100 μg/mL were prepared for the calibration curve and analysed concurrently for each 96-well plate run.

### Antimicrobial testing

The following bacterial strains were tested: methicillin-resistant *Staphylococcus aureus* (MRSA) (NCTC12493), *S*. *aureus* (NCIMB 9518and ATCC9144), *Streptococcus pyogenes* (ATCC19615), *Staphylococcus epidermidis* (ATCC12228), *Acinetobacter baumannii* (NCIMB9214), *Pseudomonas aeruginosa* (NCIMB10548) and *Escherichia coli* (NCTC10418). In addition, 5 clinical isolates of PJI were also tested.

Each bacteria spp. was stored at -80°C in cryo-protective solution. Viable stocks were generated by spreading the frozen stock on brain heart infusion (BHI) agar and incubating for 24 h at 37ºC. Plates were then stored at 4ºC for no more than 2 wk. Cell cultures were prepared by inoculating a loopful of cells from an individual colony on the plates into sterile BHI broth followed by static incubation for 24 h at 37ºC; the cell concentrations of these suspension, as determined by standard dilution/plating technique, was ~10^9^ colony-forming unit (CFU)/ml for all spp. except *S*. *pyogenes*, which was~10^8^ CFU/ml. The cell suspension was diluted 1:1000 in fresh sterile BHI broth, and 20 μL of the diluted broth were added into a sterile 96-well plate. After that, each well was filled with 100 μL media of a bone cement for each day of release testing and the plate was incubated for 18-24 h at 37 ºC. On the next day, the growth in each well was evaluated visually [36]. A sufficient growth of the tested bacteria was considered as a positive result; that is, an obvious button or definite turbidity as compared with the positive and negative growth. Each data point was performed in triplicate for each individual strain on 6 individual batches of cements specimens, to determine the duration of the release media from the bone cement inhibitory activity towards bacteria growth as the day corresponding to the last daily release inhibiting bacterial growth.

The minimum inhibitory concentration (MIC) for gentamicin was determined against the different bacteria tested through use of a standard MIC broth dilution protocol [36].

The expected antimicrobial activity against each strain was determined as the first day corresponding to a GEN concentration below MIC.

### Cytotoxicity testing

Saos-2 human osteosarcoma osteoblast-like cells (ATCC HTB-85) were used for cytocompatibility testing. Saos-2 were cultured in RPMI-1640 medium supplemented with fetal bovine serum (10% v/v) and 1% v/v of a solution of penicillin (5000 U/mL)/streptomycin (5000 mg/mL). Cells were incubated at 37°C in humidified atmosphere with 5% CO_2_. Cells were grown until ~70% confluence, washed twice with sterile PBS, and detached with trypsin, and then, osteoblast cells were counted (using Trypan Blue to differentiate between viable and nonviable cells). The cement specimens were discs (diameter and height = 10 mm and 5mm, respectively). For each of the cements, 6 specimens were tested; when the bone cements were mixed with nanoparticles these were independent batches.

#### MTT

Vybrant MTT Cell Proliferation Assay Kit (V-13154) (Thermofisher scientific, UK) was used for the determination of the number of viable cells. In a 24-well plate, each bone cement sample was incubated in 1 mL growth media inoculated with approximately 60000 cells/well for up to 7 d at 37°C in humidified atmosphere with 5% CO_2_. MTT test was done after 1, 2, 4 and 7 d of incubation. At each time point, the medium present in the well was taken off and replaced with 1 mL of fresh medium (phenol red-free). 20 μL of MTT reagent (5 mg/mL in PBS) were added to each well and the plate was incubated for 24 h at 37°C in humidified atmosphere with 5% CO_2_. After this, 900 mL of the media were removed from each of the wells, and 150 μL of dimethyl sulfoxide (DMSO) were added to each well and the plates were incubated for further 10 min. 200 μL of solution containing the dissolved formazan was put into another 96-well plate and absorbance determined using a spectrophotometer (Tecan Infinite F50, Austria) at 560 nm.

The media in each well was replaced with fresh media (1 mL of RPMI-1640 medium) warmed up to 37ºC on day 4, in order to supply the cells with nutrients before testing. Replacing media is only needed for the time point day 7, because the previous points (day 1, 2, 4) were assessed before media change.

#### LDH

In Vitro Toxicology Assay Kit, LDH based (Sigma-Aldrich, UK) was employed to determine viability of cells according to manufacturer’s protocols. Samples were prepared as described above. LDH was quantified in the media (LDH released) and after adding the cell lysis solution (LDH total). Cell viability was calculated according to the following equation:

$$Viability\;(\%)=\frac{\left(total\;LDH-LDH\;released\right)}{total\;LDH}*100\%$$ (1)

Total and released LDH were determined as OD, at 490 nm, after correcting for the reading from the negative control.

#### Calcium production assay-Alizarin red

For mineralisation, the growth medium was supplemented with 50 μM ascorbic acid-2-phosphate and 7.5 mM β-glycerophosphate. In a 24-well plate, each cement specimen was incubated in 1 mL growth media and inoculated with approximately 60000 cells/well for 21 d at 37°C in humidified atmosphere with 5% CO_2_ replacing media every 3-4 days On day 21, the medium present in each well was removed and replaced with 1 mL of glutaraldehyde 10% (v/v) (Sigma-Aldrich, UK) and the plates were incubated for 15 min and washed with deionized water three times. 1 mL of Alizarin Red S 1% (w/v) (Sigma-Aldrich, UK) was added to each well and the plates were incubated for 20 min. After washing four times with deionized water, 1 mL of acetic acid 10% (v/v) was added to each well and the plates were incubated for 30 min. After this, 200 μL of the solution were put in another 96-well plate and, then, analysed using a spectrophotometer (Tecan Infinite F50, Austria), at 450 nm [37].

#### Fluorescence imaging

Fluorescence imaging was done using specimens of Cemex Genta and Cemex-NP-GEN. Human Saos-2 cells (4x10^4^ cells/well) were seeded directly on glass coverslips in 6-well plates and cultured in RPMI 1640 medium containing 10% FBS and 1% penicillin streptomycin at 37°C in a humidified 5% CO_2_ atmosphere for 24 h. After cell attachment, the medium was replaced with 3 mL of the cement solutes obtained after incubation of the cement specimen for 24 h in RPMI 1640 medium containing 10% FBS and 1% penicillin streptomycin. Cells were washed thoroughly three times in PBS 24 h later.

The viability of cells was assessed using simultaneous fluorescence staining of viable and dead cells. Briefly, for the staining of the viable and dead cell and nuclei, cells were incubated with calcein-AM, propidium iodide (Sigma-Aldrich, St. Louis, MO, USA), and trihydrochloride Hoechst 33342 (Thermo Fisher Scientific, Eugene, OR, USA), respectively. After washing the cells with PBS, 3 mL of staining solution was added to each well (0.1% w/v propidium, 0.2% w/v calcein, 5 μg/mL Hoechst in PBS). Then, the cells were incubated at 37°C for 30 min, and the stain was removed from each, and cells were washed with PBS immediately before imaging.

A confocal microscopy (Zeiss, Oberkochen, Germany) was used for visualization of the staining, with the magnifying glasses used being 10X and 64X.

For the staining of the F-actin cytoskeleton and nuclei, the cells were fixed with 4% paraformaldehyde in PBS and permeabilized with 0.1% Triton-X-100.

Observation of cell morphology necessitated fluorescent dyes for cell staining. Thus, cells were incubated with tetramethyl rhodamine B isothiocyanate-conjugated phalloidin (Sigma-Aldrich, St. Louis, MO, USA) and trihydrochloride Hoechst 33342 (Thermo Fisher Scientific, Eugene, OR, USA), respectively. 3 mL of staining solution was added to each well (50 μg/mL fluorescent phalloidin conjugate, 5 μg/mL Hoechst in PBS). Then, the cells were incubated at 37°C for 30 min, and the stain was removed from each and cells were washed with PBS immediately before imaging.

A confocal microscopy (Zeiss, Oberkochen, Germany) was used for visualization of the staining, with the magnifying glasses used being 10X and 64X.

### Mechanical testing

Compression tests were conducted in accordance with ISO 5833 [33] using cylindrical specimens (diameter and height = 6 mm and 12 mm, respectively) and a materials testing machine (Zwick Roell ProLine table-top Z050/Z100) equipped with a dedicated software package (TestXpert II software, Zwick Testing Machines, Herefordshire, UK) at a crosshead speed of 20 mm/min. Compressive strength was determined both before and after the test specimens were aged in PBS, at 37°C, for 3 mo.

Four-point bending tests were conducted in accordance with ISO 5833 [33] using rectangular specimens (length, width, and thickness = 75 mm, 10 mm, and 3.3 mm, respectively) and the aforementioned materials testing machine and software package, at a cross-head displacement rate of 5 mm/min. Bending strength and bending modulus were determined.

Fracture toughness tests were conducted in accordance with ISO 13586 ISO13586:2000 [38] using rectangular specimens (length, width, and thickness = 45.0 ± 0.1 mm, 10.0 ± 0.1 mm, and 3.3 ± 0.1 mm, respectively) with a sharp chevron notch (5.5 ± 0.5 deep) cut into the center of one of the long sides of the specimen using a sharp razor blade. The specimen was loaded at the center of the unnotched long face, in three-point bend mode (distance between the support rollers = 40 mm) using the aforementioned materials testing machine and software package, at a cross-head displacement rate of 5 mm/min.

For each combination of cement and mechanical test, 5 specimens were tested; when the bone cements were mixed with nanoparticles these were independent batches.

### PBS uptake testing

The bone cement cylindrical specimens were incubated in 3 mL PBS at 37°C for 35 d; for the first 2 wk, the samples were weighed daily; and, after that, they were weighed every 3 d [34]. At each time-point, the PBS uptake of a specimen was calculated by dividing its mass gain by its initial mass. For each cement, 3 specimens were tested; when the bone cements were mixed with nanoparticles these were independent batches.

### Statistical analysis

Each of the quantitative results is given as mean ± standard deviation. The hypothesis of normal distribution of the results was tested using the Shapiro-Wilk test, with significance denoted when p < 0.05. Test of significance was conducted using one-way analysis of variance (ANOVA) and significance was denoted if p < 0.05. All analyses were run using a commercially available software package (IBM SPSS Statistics for Windows, version 21 (IBM Corp., Armonk, NY, USA).

## Results

### Bone cement settling time

**Fig 1:**
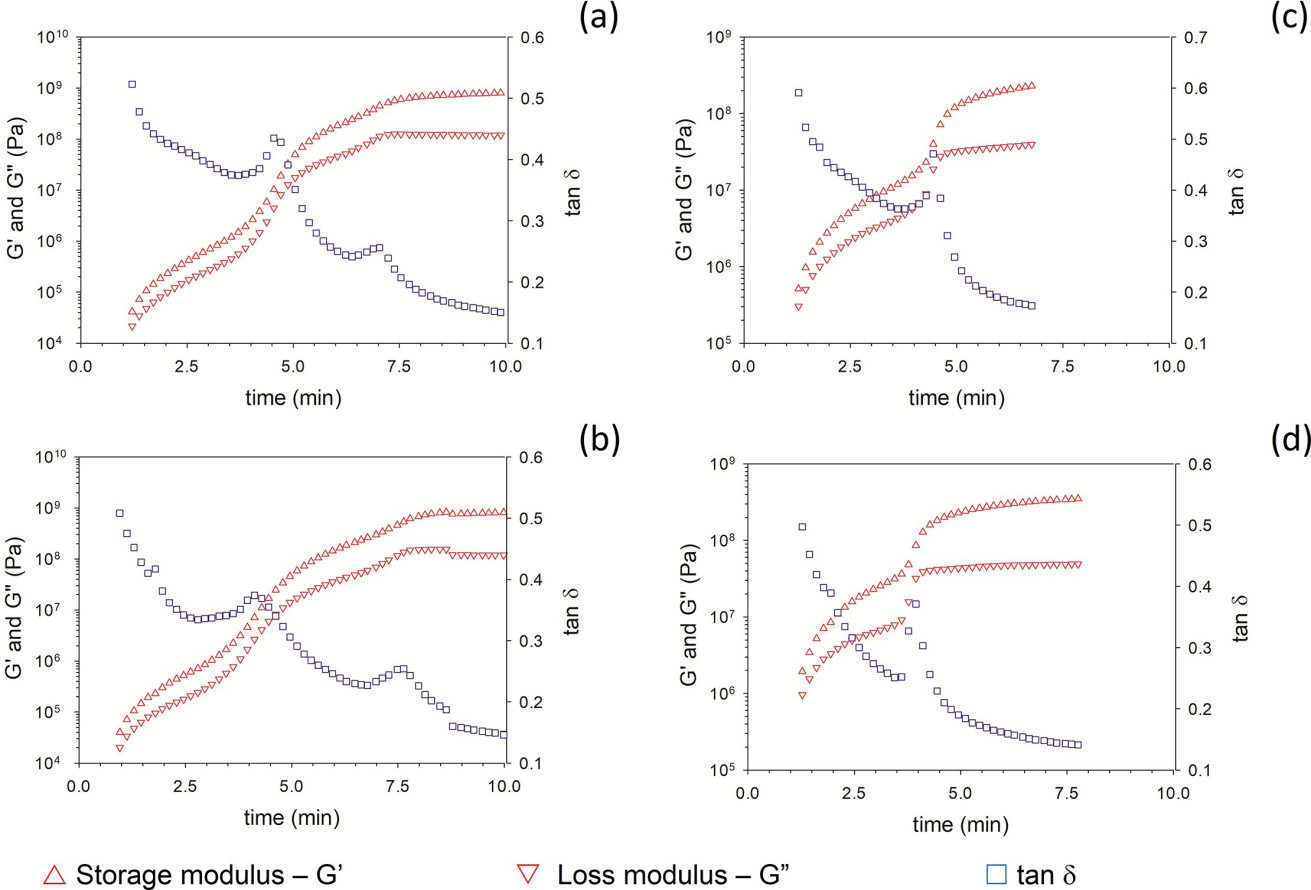
Sample rheological test results for Palacos R+G (a), Palacos-NP-GEN (b), Cemex Genta (c) and Cemex-NP-GEN (d).

For both Cemex and Palacos R, incorporation of NP-GEN led to an insignificant decrease in setting time (p>0.05) (from ~4.5±0.1 min to ~ 4.3±0.1 min) (**Fig 1**).

### Gentamicin release profile

**Fig 2:**
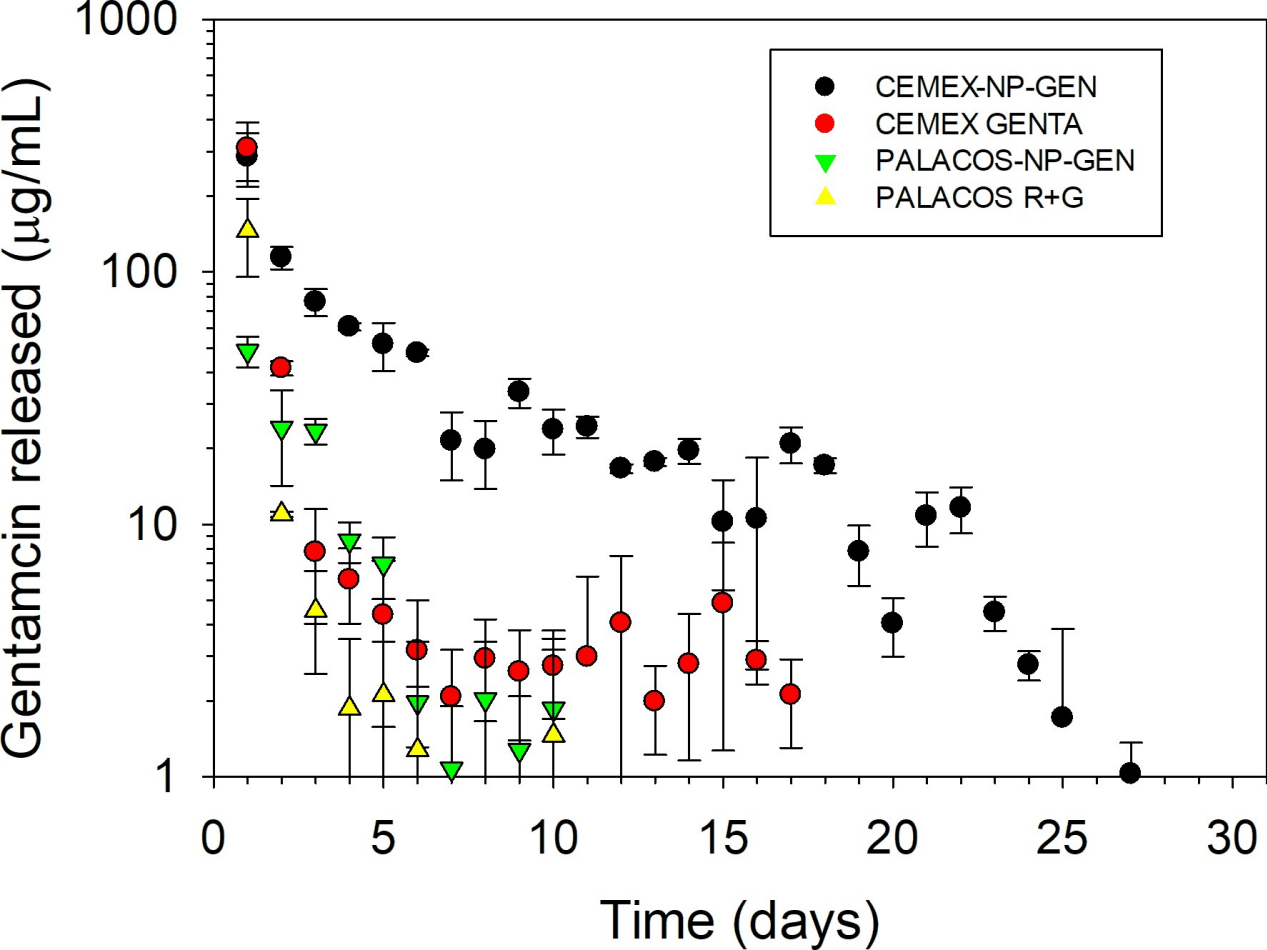
Summary of the gentamicin release results (n=6±SD).

For Cemex and Palacos R, 1) the concentration of GEN released in the media had a burst profile (**Fig 2)** with concentration of ~315 and 150 μg/mL, respectively, after 1 d that dropped at least an order of magnitude after 2 d; and 2) GEN release continued for about 1 wk and 2 wk in the case of Palacos R+G and Cemex Genta, respectively (**Fig 2)**.

When NP-GEN was added to the bone cement, the release of GEN from Cemex-NP-GEN exhibited the same concentration after 1 d but the subsequent decrease in antibiotic released was less pronounced and gentamicin was released for ~27 d.

A lower concentration of GEN was released from Palacos-NP-GEN than Palacos R+G after 1 d, but the release was not prolonged as GEN concentration fell below 1 μg/mL after 10 d.

Cumulative GEN release from Cemex-NP-GEN (~26%) was significantly higher than from Cemex Genta (~10%); while, for both Palacos-NP-GEN and Palacos R+G, cumulatively, only ~9% of the initially added GEN was released.

### Antimicrobial analysis

MICs for the strains ranged between 16 μg/mL for MRSA and 1 μg/mL for *S*. *epidermidis* (**Table 2** and **Table 3)**.

**Table 2:** Gentamicin MIC of catalogues strains tested.

*Strain*	MIC (μg/mL)
*S*. *aureus* NCIMB 9518	8
*S*. *aureus* ATCC 9144	1
*S*. *epidermidis* ATCC12228	1
MRSA NCTC12493	15.5
*S*. *pyogenes* ATCC12344	8
*E*. *coli* NCTC14418	8
*P*. *aeruginosa* PA01	8
*A*. *baumannii* NCIMB 9214	2

**Table 3:** Gentamicin MIC of clinical isolates tested.

*Strain*	MIC (μg/mL)
*S*. *pneumonia* 59413	8
*P*. *aeruginosa* 59224	8
*S*. *epidermidis* 59174	4
*E*. *coli* 59284	8

**Fig 3:**
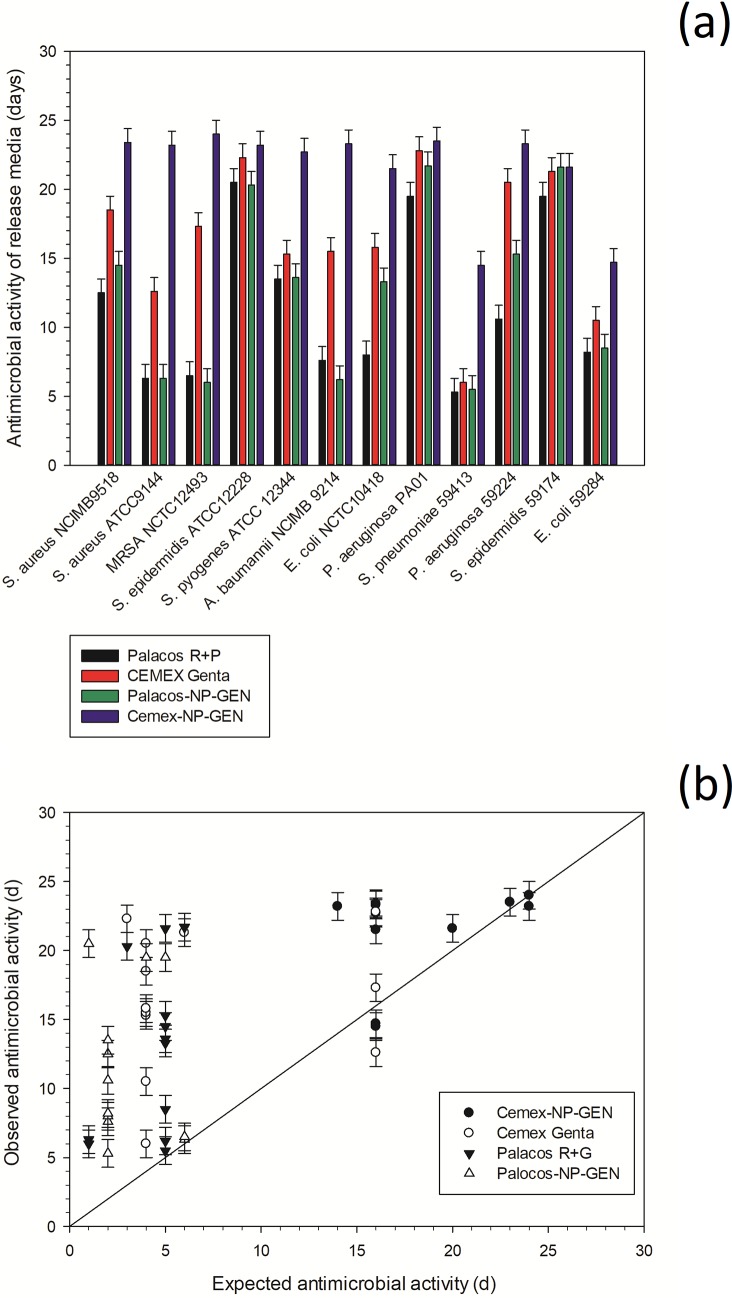
Summary of the antimicrobial test results (n=6±SD) (a) and expected versus observed antimicrobial activity (b).

Cemex-NP-GEN showed longer duration of bacterial growth inhibition compared to Cemex Genta (**Fig 3A**).

For most of the bacterial species tested, Palacos-NP-GEN did not show longer antimicrobial activity than Palacos R+G.

The expected duration of antimicrobial activity for each strain was determined as the first day when the concentration of GEN in the release media was below the MIC. Generally, the observed antimicrobial activity was longer than expected (if only GEN concentration was taken into account), particularly for strains with greater MIC (**Fig 3B**).

### Cytotoxicity analysis

#### MTT assay

**Fig 4:**
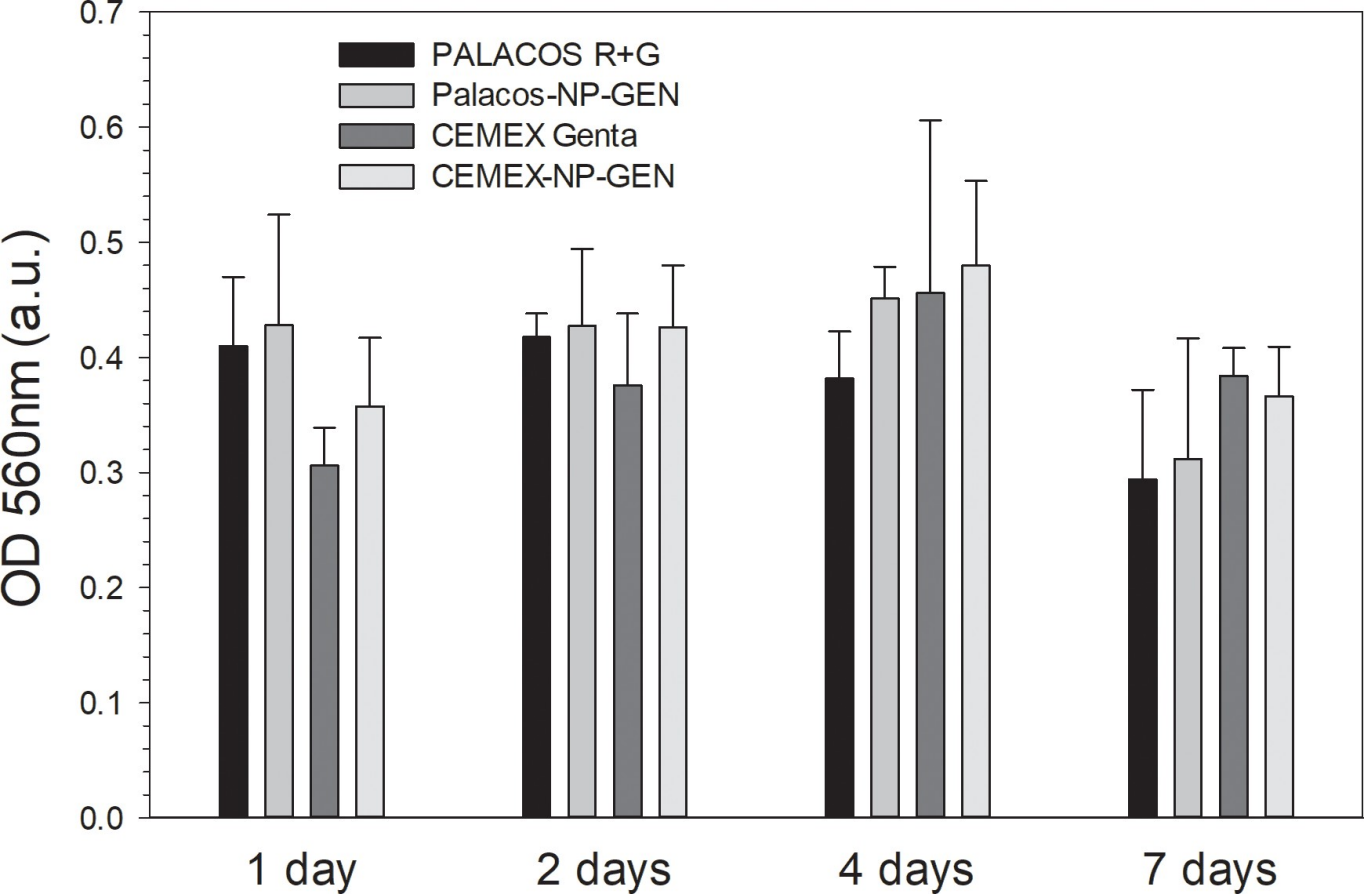
Summary of mitochondrial activity of osteoblasts exposed to bone cements assessed through MTT (n=6±SD).

After each of the time points (1, 2, 4 and 7 d), mitochondrial activity of osteoblast cells grown on bone cements samples did not differ between Palacos R+G and Palacos-NP-GEN (p>0.05) nor between Cemex Genta and Cemex-NP-GEN (p>0.05) (**Fig 4**). However, after 1 d, the activity of Cemex was lower than that of Palacos R (p<0.05).

#### LDH assay

**Fig 5:**
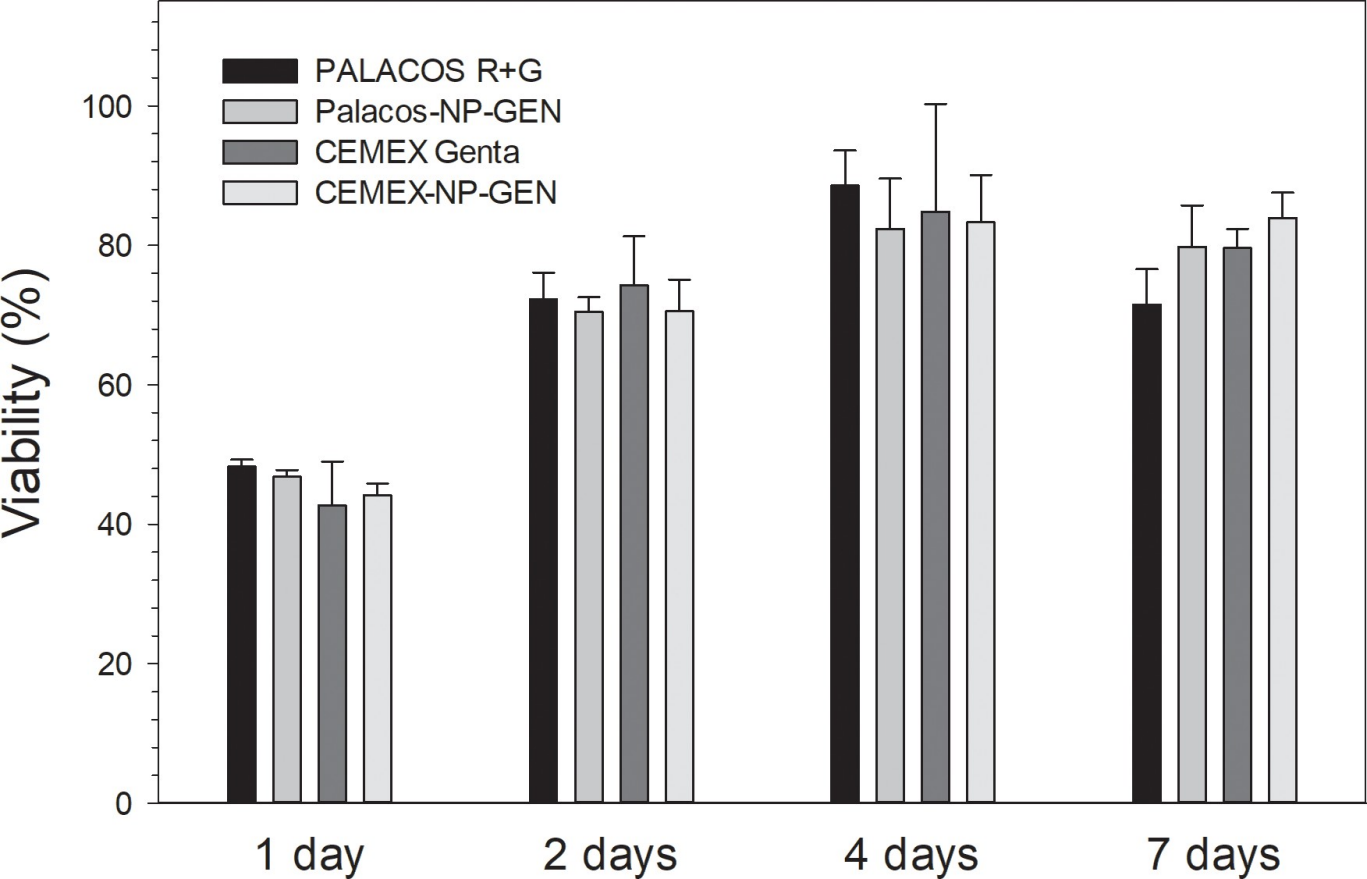
Summary of cytocompatibility test results, assessed through LDH assay: (n=6±SD).

After each of the time points (1, 2, 4 and 7 d), the viability of human osteoblast cells exposed to Palacos or Cemex bone cement was not affected by the addition of either GEN or NP-GEN (p> 0.05) (**Fig 5**).

#### Alizarin red

**Fig 6:**
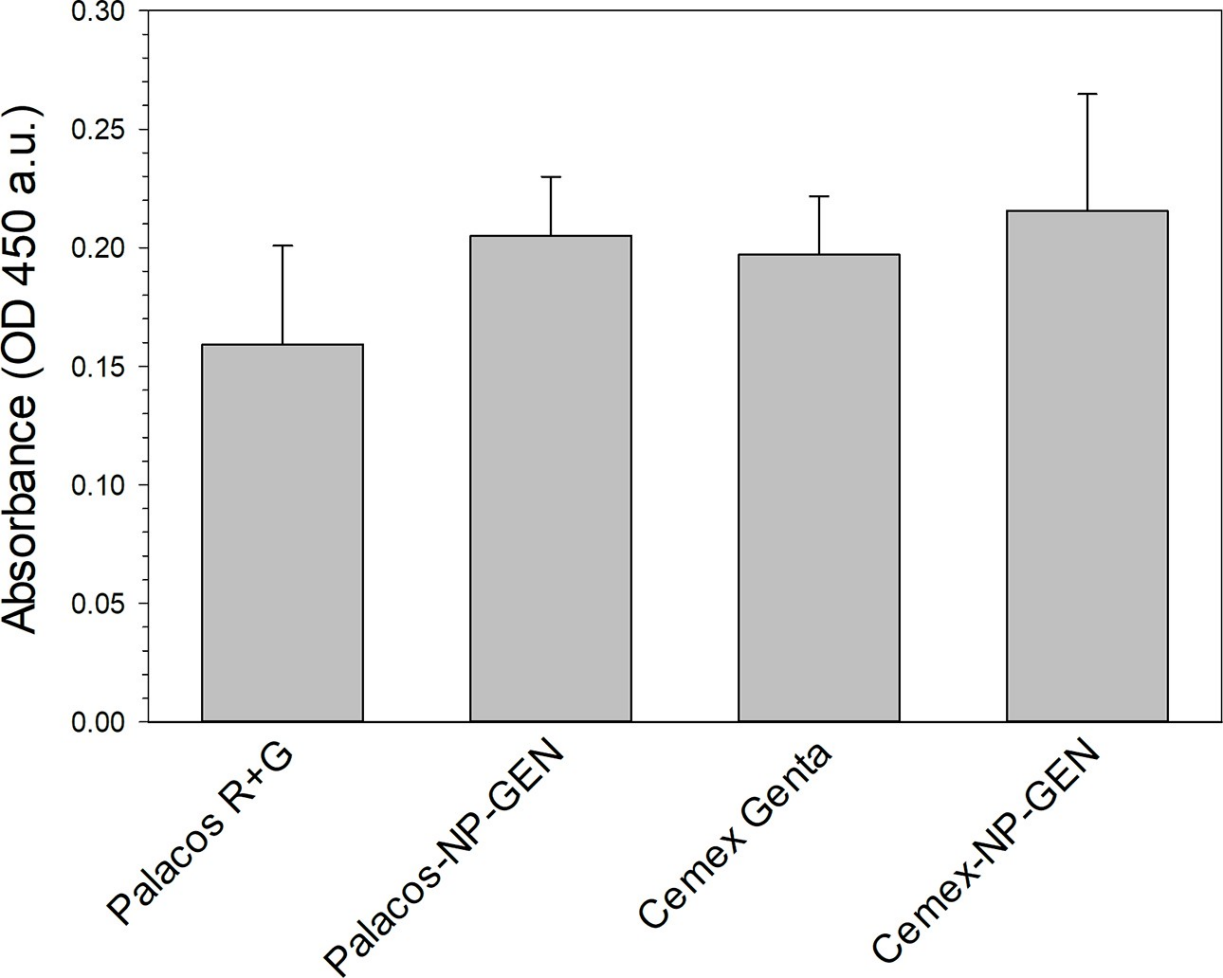
Summary of the cytocompatibility test results, assessed through Alizarin Red assay (n=6±SD).

Reduction in calcium production for osteoblasts exposed to the NP-GEN loaded cements (Palacos-NP-GEN and Cemex-NP-GEN) was not significant compared to their commercial counterparts (p>0.05) (Palacos R+G and Cemex Genta, respectively) (**Fig 6**).

#### Fluorescence images

**Fig 7:**
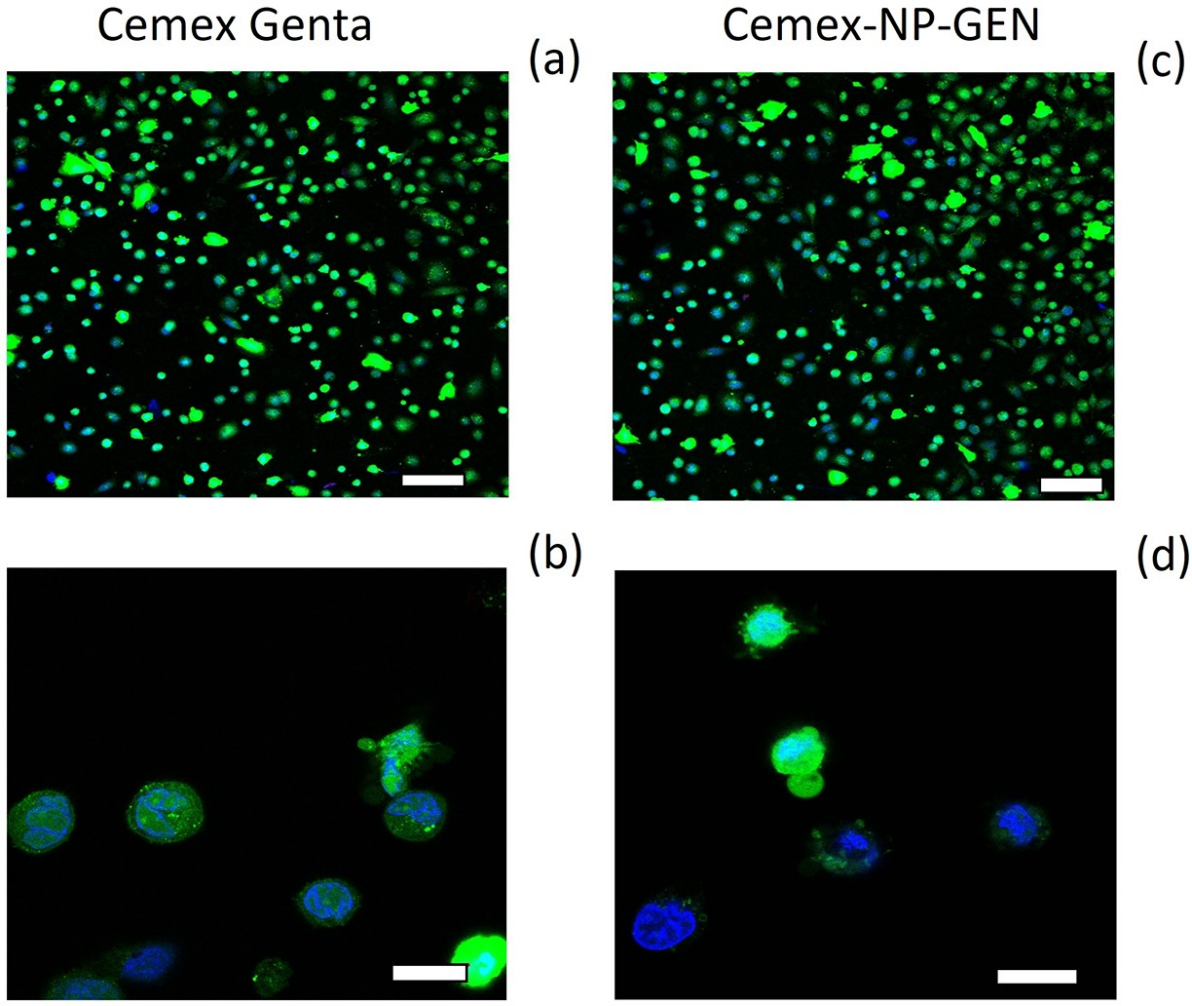
Summary of the images of the live cells (green color), dead cells (red color), and cell nuclei (blue color). (Top panels: 100 μm bar, bottom panels: 20 μm bar).

Live and dead fluorescent images of osteoblast cells for Cemex Genta and Cemex-NP-GEN appeared to be similar (**Fig 7**).

**Fig 8:**
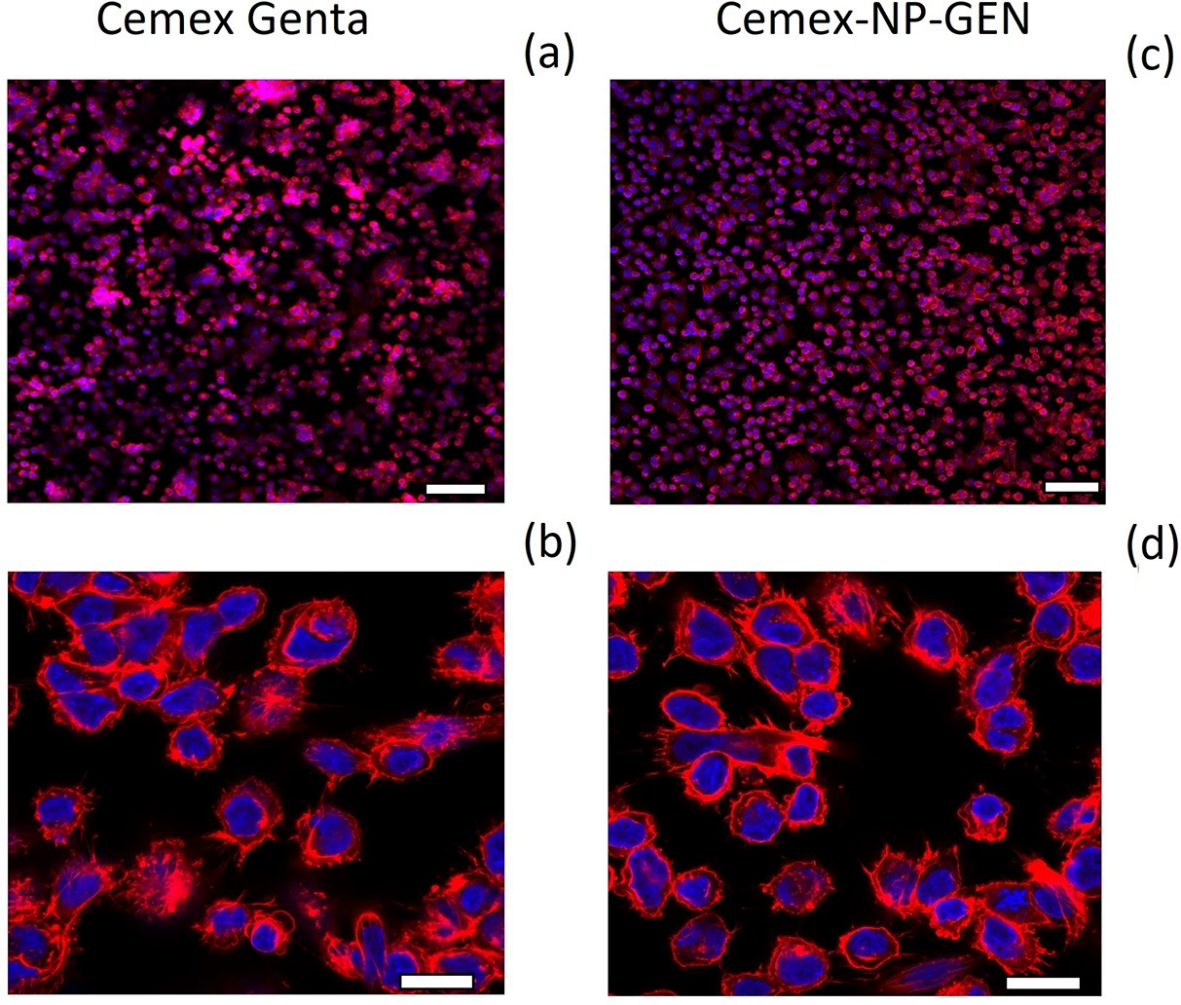
Summary of the images of the actin/dapi filaments (red color) and the cell nuclei (blue color). (Top panel: 100 μm bar, bottom panel: 20 μm bar).

Actin staining suggests bone cement specimens were a substrate for which the cells have some, albeit poor, compatibility (**Fig 8)**. Osteoblast cells are totally spreading, especially considering the number of filopodia apparent at the higher magnification (**Fig 8B** and **8D**). The normal trapezoidal morphology of osteoblast-like cells is missing, as are the stress filaments characteristic of well-spread osteoblasts.

### Mechanical properties

**Fig 9:**
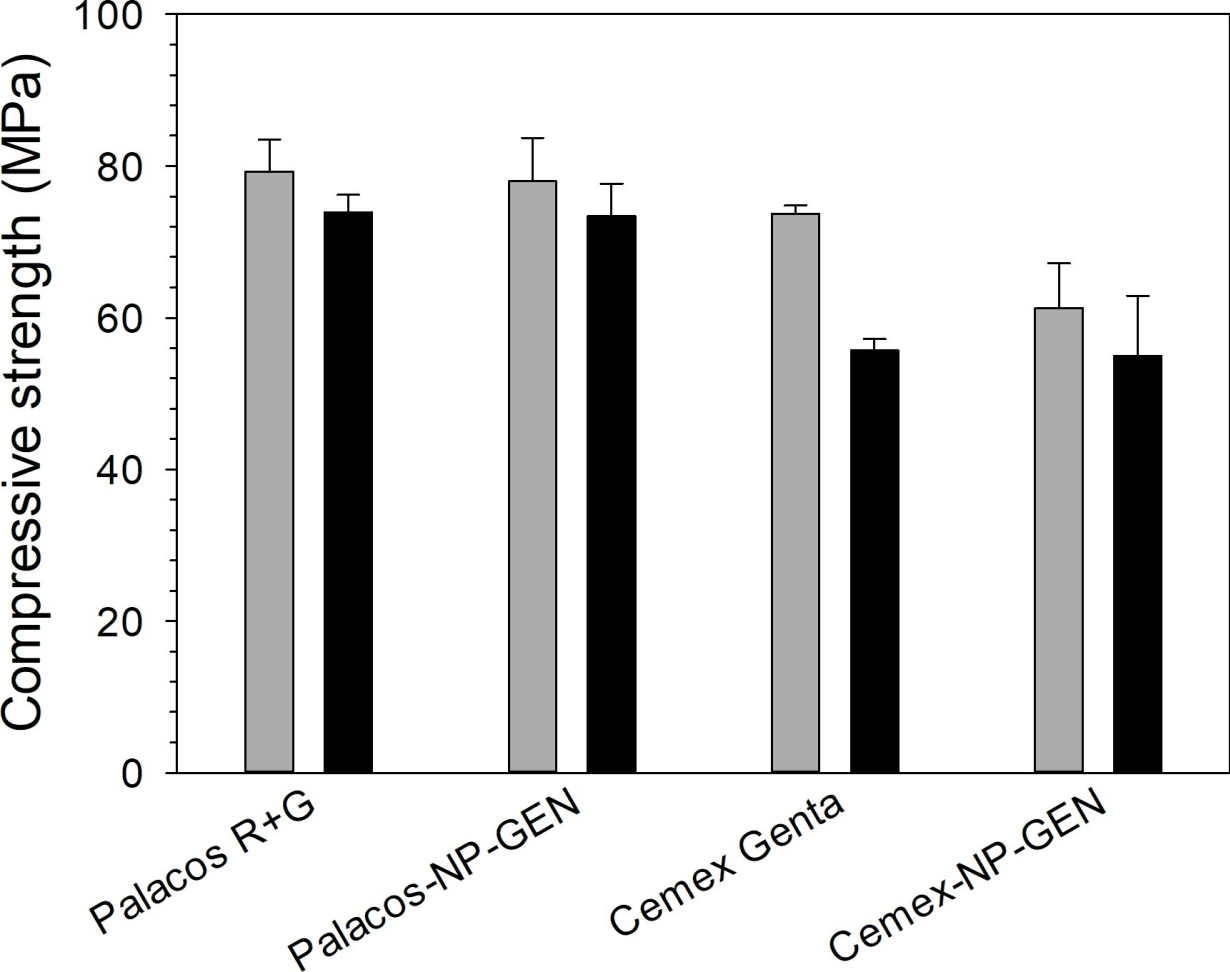
Summary of compressive strength bone cement bone cements before (grey column) and after aging in PBS, at 37 ºC, for 3 mo (black columns) (n=5±SD).

NP-GEN-containing bone cements had similar compressive strength to the commercial ones both before and after ageing of the specimens (p>0.05) (**Fig 9**). Palacos cements had higher compressive strength than Cemex strength (p>0.05). Aging of test specimens resulted in decrease of compressive strength of Cemex specimens (p<0.05) but not for Palacos specimens (p>0.05).

Cemex-NP-GEN showed similar bending strength, bending modules, and fracture toughness to the corresponding values for Cemex Genta (p>0.6) (**Table 4**).

**Table 4:** Mechanical properties of the Cemex bone cements.

	Bending strength (MPa)	Bending modulus (MPa)	Fracture toughness (MPa√m)
Cemex Genta	54.3 ± 2.0	2901 ± 62	2.4 ± 0.5
Cemex-NP-GEN	51.2 ± 4.1	2964 ± 101	2.2 ± 0.3

### PBS uptake testing

**Fig 10:**
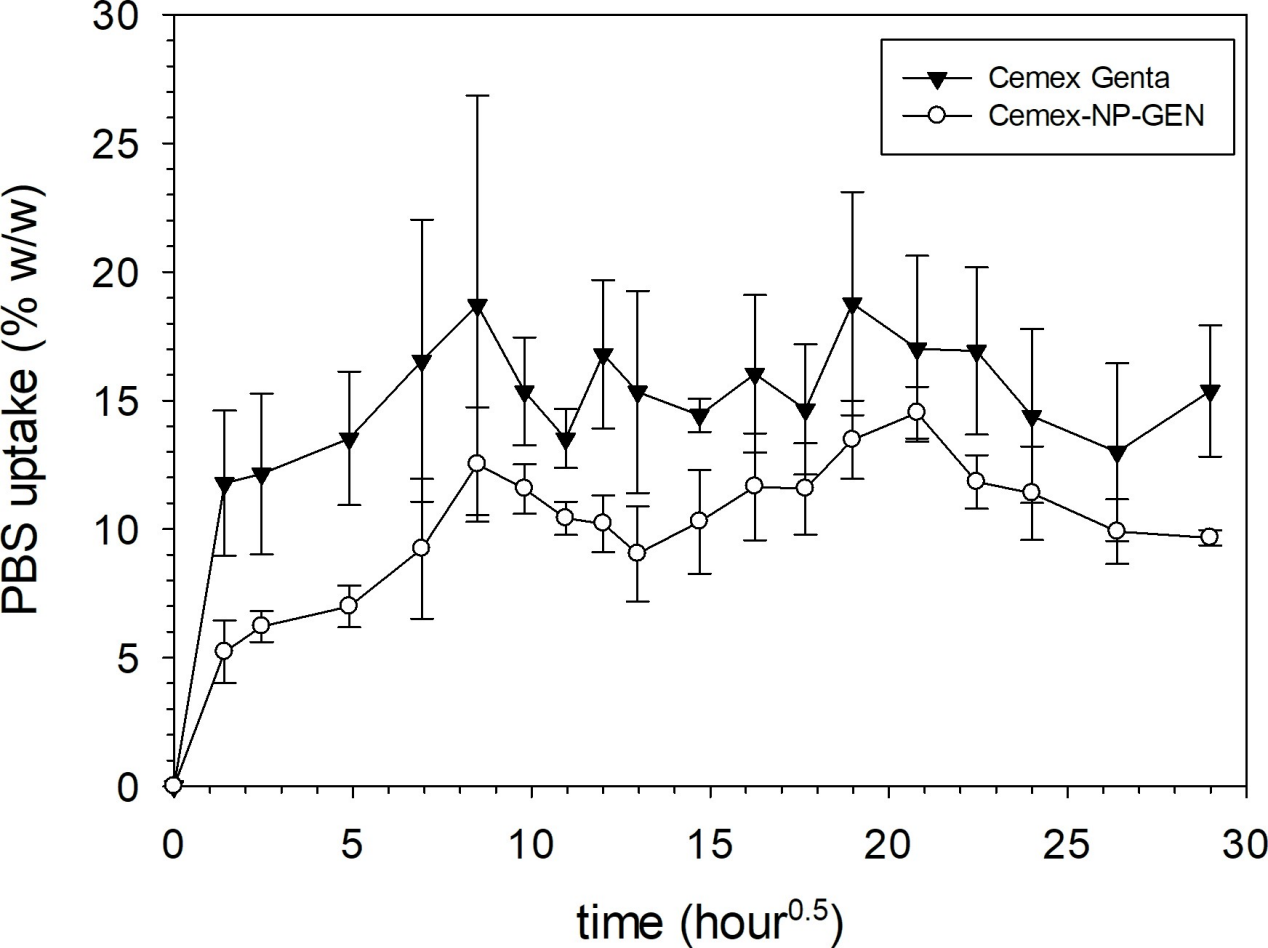
Summary of the PBS uptake test results for different types of gentamicin containing bone cements after incubation in PBS buffer, pH 7.4 (n=3±SD).

Overall, PBS uptake by Cemex-NP-GEN specimens was comparable to that by Cemex Genta specimens up to 35 d (p>0.05) (**Fig 10**).

## Discussion

TJRs are performed on patients affected by end stage osteoarthritis (OA). These procedures are conducted with increasing frequency because of the increasing incidence of OA that is driven by the ageing population and obesity. A downside of TJR is that obesity and age are also risk factors associated to PJI [39]; ALBCs are routinely used to prevent and treat such occurrences. Unfortunately, PJI can establish well after the bone cement has ceased releasing antibiotic; hence, novel technologies capable of prolonging the release of antibiotics from bone cements are urgently needed [40]. Silica nanoparticles loaded with gentamicin through LbL have shown ability to sustain antibiotic release for ~4 wk [31]. The purpose of the present study was to determine whether such nanocarriers could be mixed in commercial bone cements, resulting in sustained antibiotic release, without negative impact on other properties. For this purpose, we used bone cements brands produced by two different manufacturers (characterised by different composition/concentration of radio-opacifier, polymer used and amount of antibiotic).

Settling time is a critical parameter for bone cement use during application and after patient recovery, because it determines the time available to the surgeon to apply the bone cement and the time needed to develop the final mechanical properties of the cement. NP-GEN-containing bone cements (Cemex-NP-GEN and Palacos R-NP-GEN) were not inferior in terms of settling time (**Fig 1**) than the equivalent commercial formulation (Cemex Genta and Palacor R+G, respectively). The setting time depends on the polymerisation of methyl methacrylate and PMMA and, because of the significant amount of granular material present in the bone cement as radio opacifiers, the addition of the required amount of NP-GEN did not influence the kinetic of polymerisation. The present G’ and G’’ profiles were comparable to those presented by others for PMMA bone cements [34, 41].

The concentration of NP-GEN used in the bone cement was calculated in order to have equivalent amount of gentamicin to the commercial cements. Cemex-NP-GEN exhibited prolonged release, the reason for this behaviour being that the antibiotic needs first to be released from the coating on the silica NP before it can migrate through the bone cement matrix. Gentamicin exhibits high diffusivity through PMMA bone cements, resulting in burst release from commercial formulations, but the antibiotic is gradually made available and, thus, the release occurs over a longer period of time. The use of NP-GEN did not extend the release of gentamicin in Palacos as these cements are prepared with smaller amount of gentamicin (**Table 1**) and the antibiotic concentration fell below detection limits before the effect of the nanocarriers could be observed. Moreover, the enhanced total amount of gentamicin released from the bone cement (26% of GEN) by 2-3 folds compared to the commercial formulation noticed in Cemex-NP-GEN could be the result of the homogenous distribution of NP-GEN in the bone cement matrix, which may lead to the formation of nano-network channels to facilitate the diffusion of GEN [42].

The prolonged antimicrobial activity against rapidly proliferating pathogens of Cemex-NP-GEN compared to Cemex Genta (**Fig 3**) is the result of the antibiotic concentration remaining above MIC for longer periods of time. As Palacos-NP-GEN and Palacos R+G exhibited similar release profiles, the antimicrobial activity was not expected to be different. Antimicrobial activity against pathogens routinely associated to PJI [43, 44] was due to the presence of GEN above MIC. Moreover, the different inhibitory activity determined on the variety of pathogens employed depends on the bacteria susceptibility to antibiotic; that is, not species specific, but, also, strain dependent. Moreover, we employed planktonic cells that are normally more susceptible to antimicrobial compounds that their adhering (biofilms) counterparts [45]. Longer antimicrobial inhibition than expected (simply based on gentamicin concentrations) were observed here (**Fig 3B**); which allowed us to hypothesize that unreacted monomer was also released from bone cement specimens. The presence of unreacted methyl methacrylate is a well-known [46] phenomenon and such a compound is likely to synergistically contribute to the antimicrobial activity exhibited by the release media. The release of methyl methacrylate is also likely to decrease with time (similarly to antibiotics), thus, the effect would terminate and the observed duration of antimicrobial activity on strains with low MIC should be closer to the expected. The greater disparity between observed and expected antimicrobial duration for Palacos than Cemex could be attributed to low GEN concentration measured in the release media (and corresponding short expected antimicrobial duration). Remarkably, the antimicrobial duration in Cemex-NP-GEN was nearly as expected for strains with low MIC; these would be inhibited for longer when methyl methacrylate release had already ended, leaving of GEN as inhibitory compound in the release media.

Successful TJR requires osseointegration of the device through osteoblasts growth on the bone cements. This material does not represent an optimal substrate for cells growth [47, 48], this was also visible in the acting staining images (**Figs 7** and **8**) and in the LDH assay results (**Fig 5**); but bone cements cytocompatibility is sufficient as they are routinely employed. Our experiments were, therefore, interested in demonstrating the non-inferiority of the bone cement with mixed NP-GEN compared to the corresponding commercial formulation using biochemical assays (**Figs 4, 5** and **6**) and imaging (**Figs 7** and **8**). No differences were caused by NPs addition in any of the tests as silica NPs are known to be highly biocompatible and also the LbL coating is made with biocompatible polyelectrolytes.

Compressive strength (**Fig 9**), bending strength, bending modulus, and fracture toughness (**Table 4**) were not affected by the addition of NP-GEN as the amount of nanocarriers mixed is minimal compared to the already present non-polymeric constituents. Palacos has a higher compressive strength than Cemex because of the different composition that also justifies the different behaviour after aging (Cemex decreasing compressive strength after 3 mo while Palacos retaining the compressive strength exhibited after preparation). Bending strength, bending modulus, and fracture toughness were only tested for Cemex Genta and Cemex-NP-GEN because the use of nanocarriers in Palacos cement was not able to prolong the release of GEN (**Fig 2**).

Cemex-NP-GEN has a higher PBS intake than Cemex Genta (**Fig 10**) in the first hours of contact with the liquid, after which the two bone cements absorbed similar amount of PBS. It is possible that the NP-GEN induced the formation of more channels during polymerisation which allowed more PBS to be adsorbed. Moreover, the higher PBS intake may also contribute to the increased cumulative release of GEN.

Two nanostructure-based gentamicin-loaded carriers have been studied to improve the release profile of gentamicin from ALBCs [42, 49]. Shen et al. (2016) [42] used mesoporous silica nanoparticles (MSN) to improve gentamicin release from PMMA bone cement that continued for up to 80 days, where 60% of gentamicin was released. However, the concentration of released gentamicin from MSN or the loading efficiency of MSN were not stated in the report. In the present study, the loading efficiency of GEN in the NP-GEN was ~30% (w/w), which is lower than that in MSN [42]. This is because MSNs have larger size (100–300 nm) with pores in the range 5–30 nm allowing higher gentamicin loading inside the pores compared to NP-GEN. Ayre et al. [49] loaded gentamicin into liposomes and antibiotic release from loaded cement continued for up to 30 d, with 22% of the loaded antibiotic released. These results are similar results to the present one. However, the addition of liposomes to bone cement did significantly impact the compressive strength of the cement, whereas, in the present study, addition of NP-GEN did not do so.

For the determination of setting time, dynamic rheology was chosen over standard method (ISO 5833 and ASTM F451) in order to provide a more objective result (as it does not require operator decision) and more reproducible as ISO5833 results depends on, for example, the brand of glove used [50]. Different protocols are used to evaluate the antimicrobial properties of bone cements in literature beside counting surviving bacteria through plating. For example, a high throughput technique based on the duration of apparent lag phase has been developed by Bechert et al. (2000)[51] while agar diffusion test to evaluate the antimicrobial properties of liposomal cement formulation [49]. Hence, there is not a standardised approach to estimating antimicrobial activity. The protocol that we used has the advantage of allowing screening numerous strains using the same specimens, which is not possible with diffusion test or any other of the methodologies mentioned. A wide range of cytotoxicity assays (MTT, LDH, Alizarin red and microscopy image) was employed in this study to provide a more comprehensive validation of the drug delivery system as each test assesses a specific variable (such as MTT mitochondrial activity, LDH cell integrity, Alizarin red calcium production and microscopy cell morphology). Despite the need for long term in vivo trials to fully address cytocompatibility, in vitro data can provide indicative responses and, thus, the greater the number of tests the greater the number of variables evaluated.

The present study has a number of limitations. First, the study was conducted using one set of processing/fabrication conditions. It is likely that different outcomes would be obtained if different polyelectrolytes were used during LbL (for example, PBAEs synthesised with other acrylates and amine exhibiting varying zeta potential, length and kinetics of hydrolysis); Second, the ability of the released GEN to inhibit formation of biofilms (a phenomenon that has been widely reported to be implicated in PJI) [52, 53] was not investigated.

## Conclusion

Lbl-coated silica nanoparticles in which gentamicin sulphate was incorporated have been successfully mixed with the powder of a PMMA bone cement (NP-GEN cement). Compared to a commercial counterpart cement, NP-GEN cement has comparable setting time (~4.5 min), displayed GEN release that was more gradual and prolonged (burst phase duration of ~7 d versus ~1 d and exhaustion after ~22 d versus ~4 d), demonstrated significantly higher efficacy of the eluted gentamicin against an assortment of bacterial strains, including those from clinical isolates from infected TJRs (for example, Cemex-NP-GEN and Cemex Genta inhibited growth of each of the bacterial strains from PJI isolates for ~19 ± 4 d and ~14 ± 8 d, respectively), showed cytocompatibility (towards Saos-2 human osteosarcoma osteoblast-like cells), and have comparable compressive strength (75 ± 4 MPa and 78 ± 2 before and after aging in PBS, at 37 ^o^C, for 3 mo), 4-point bend strength and modulus (52 ± 2 MPa and 2935 ± 15 MPa, respectively), fracture toughness (2.3 ± 0.2 MPa√m), and PBS intake (a steady-state value of ~11 wt./wt.%). Thus, NP-GEN bone cement may have a role in preventing or treating PJI.

## Supporting information

S1 Table Rheological data (G', G'' and tan delta) vs time for bone cements. (XLSX)

S2 Table GEN release. (XLSX)

S3 Table Length antimicrobial activity. (XLSX)

S4 Table MTT, LDH and Alizarin Red results. (XLSX)

S5 Table Compressive strength. (XLSX)

S6 Table PBS uptake. (XLSX)
